# BCFtools/liftover: an accurate and comprehensive tool to convert genetic variants across genome assemblies

**DOI:** 10.1093/bioinformatics/btae038

**Published:** 2024-01-23

**Authors:** Giulio Genovese, Nicole B Rockweiler, Bryan R Gorman, Tim B Bigdeli, Michelle T Pato, Carlos N Pato, Kiku Ichihara, Steven A McCarroll

**Affiliations:** Program in Medical and Population Genetics, Broad Institute of MIT and Harvard, Cambridge, MA 02142, United States; Stanley Center, Broad Institute of MIT and Harvard, Cambridge, MA 02142, United States; Department of Genetics, Harvard Medical School, Boston, MA 02115, United States; Program in Medical and Population Genetics, Broad Institute of MIT and Harvard, Cambridge, MA 02142, United States; Stanley Center, Broad Institute of MIT and Harvard, Cambridge, MA 02142, United States; Department of Genetics, Harvard Medical School, Boston, MA 02115, United States; Center for Data and Computational Sciences, VA Boston HealthCare System, Boston, MA 02130, United States; Booz Allen Hamilton Inc, McLean, VA 22102, United States; Department of Psychiatry and Behavioral Sciences, SUNY Downstate Health Sciences University, Brooklyn, NY 11203, United States; Institute for Genomics in Health, SUNY Downstate Health Sciences University, Brooklyn, NY 11203, United States; Cooperative Studies Program, VA New York Harbor Healthcare System, Brooklyn, NY 11209, United States; Department of Psychiatry, Robert Wood Johnson Medical School, New Brunswick, NJ 08901, United States; Department of Psychiatry, Robert Wood Johnson Medical School, New Brunswick, NJ 08901, United States; Stanley Center, Broad Institute of MIT and Harvard, Cambridge, MA 02142, United States; Department of Genetics, Harvard Medical School, Boston, MA 02115, United States; Program in Medical and Population Genetics, Broad Institute of MIT and Harvard, Cambridge, MA 02142, United States; Stanley Center, Broad Institute of MIT and Harvard, Cambridge, MA 02142, United States; Department of Genetics, Harvard Medical School, Boston, MA 02115, United States

## Abstract

**Motivation:**

Many genetics studies report results tied to genomic coordinates of a legacy genome assembly. However, as assemblies are updated and improved, researchers are faced with either realigning raw sequence data using the updated coordinate system or converting legacy datasets to the updated coordinate system to be able to combine results with newer datasets. Currently available tools to perform the conversion of genetic variants have numerous shortcomings, including poor support for indels and multi-allelic variants, that lead to a higher rate of variants being dropped or incorrectly converted. As a result, many researchers continue to work with and publish using legacy genomic coordinates.

**Results:**

Here we present BCFtools/liftover, a tool to convert genomic coordinates across genome assemblies for variants encoded in the variant call format with improved support for indels represented by different reference alleles across genome assemblies and full support for multi-allelic variants. It further supports variant annotation fields updates whenever the reference allele changes across genome assemblies. The tool has the lowest rate of variants being dropped with an order of magnitude less indels dropped or incorrectly converted and is an order of magnitude faster than other tools typically used for the same task. It is particularly suited for converting variant callsets from large cohorts to novel telomere-to-telomere assemblies as well as summary statistics from genome-wide association studies tied to legacy genome assemblies.

**Availability and implementation:**

The tool is written in C and freely available under the MIT open source license as a BCFtools plugin available at http://github.com/freeseek/score.

## 1 Introduction

As sequencing technologies and analysis tools improve, the original first draft of the human genome (International Human Genome Sequencing Consortium 2004) has undergone numerous updates (Church *et al.* 2011, Schneider *et al.* 2017) and more recently the scalability of long-read sequencing technologies allowed the first telomere-to-telomere assembly (T2T-CHM13v2.0) of a haploid human genome without gaps (Aganezov *et al.* 2022, Nurk *et al.* 2022, Rhie *et al.* 2023). The reality of multiple genome assemblies being used means that researchers and clinical laboratories provide results tied to different coordinate systems, most commonly with the legacy GRCh37 human genome assembly being often favored over the updated GRCh38 assembly (Lansdon *et al.* 2021, Li *et al.* 2021).

The need to convert variant genomic coordinates routinely arises when performing meta-analyses and computing polygenic scores starting from genome-wide association studies (GWAS) summary statistics tied to legacy genome assemblies. Usage of summary statistics commonly requires time-consuming harmonization steps to run secondary analyses (Murphy *et al.* 2021, Matushyn *et al.* 2022), something that would not be required if such datasets were available in a standardized file format and secondary analysis tools such as liftover tools were readily available and compatible with such file format.

To convert the coordinates of a genomic interval from one genome assembly to another one, can use the UCSC liftOver tool (http://genome.ucsc.edu/cgi-bin/hgLiftOver). While this approach works reasonably well for single nucleotide variants (SNVs), which can be represented by a one base pair genomic interval, a more sophisticated strategy is needed when converting indels and short tandem repeats (STRs) as different genome assemblies do not necessarily represent the same allele for a given variant.

At the time of the writing of this manuscript, to convert the coordinate system of variant call format (VCF) (Danecek *et al.* 2011) files from one genome assembly to another (most commonly from GRCh37 to GRCh38) most researchers use Picard/LiftoverVcf (http://broadinstitute.github.io/picard/) or CrossMap/VCF (Zhao *et al.* 2014), two tools that for the most part employ an approach limited to converting the genomic interval covered by the variant reference allele. As Picard/LiftoverVcf can handle SNV records for which the two genome assemblies are represented by different alleles, SNVs are dropped mostly because of genomic loci missing from one genome assembly (Ormond *et al.* 2021). However, both Picard/LiftoverVcf and CrossMap/VCF cannot handle swapping the reference and alternate alleles for indel records leading to many of these variants either being dropped or being converted incorrectly with the risk of introducing biases in downstream analyses (McLean *et al.* 2019, Lan *et al.* 2022, Weisburd *et al.* 2023). There are three VCF liftover tools that can handle reference allele differences between assemblies for indel records: (i) Transanno/liftvcf (http://github.com/informationsea/transanno), which can also deal with multi-allelic VCF records; (ii) Genozip/DVCF (Lan *et al.* 2022), included in the Genozip software compression suite (Lan *et al.* 2020; Lan 2022); and (iii) GenomeWarp (McLean *et al.* 2019), which was designed to have higher accuracy at the cost of a larger number of variants being dropped. Nevertheless, as allelic differences for indels between genome assemblies are not always handled correctly by any of the available liftover tools, indel records are always dropped or incorrectly converted at a higher rate than SNV records.

We engineered a VCF liftover tool that uses a more advanced strategy to work around allelic differences between genome assemblies with the result that indels and multi-allelic variants are handled almost as well as SNVs even when genome assemblies are represented by different alleles. When required, the tool also updates many variant annotation fields including those related to GWAS summary statistics encoded following the GWAS-VCF specification (Lyon *et al.* 2021), a more robust and efficient format than other standards being proposed (Hayhurst *et al.* 2022).

## 2 Methods

### 2.1 Definitions

When BCFtools/liftover processes indel VCF records, rather than mapping each base pair of the segment defining the region that the record might affect, it maps to the new assembly the two edge base pairs of an extended region that can be recognized as affected by the variant. Use of this extended region allows proper consideration of STR length differences across genome assemblies.

A VCF record is left-aligned if and only if its base position is smallest among all potential VCF records having the same allele length and representing the same variant. A VCF record is parsimonious if and only if the record has the shortest allele length among all VCF records representing the same variant. A VCF record is normalized if and only if it is left-aligned and parsimonious (Tan *et al.* 2015).

We introduce the definition of a maximally extended VCF record as a record for which:

1. For each pair of alleles the short allele is not identical to the prefix or the suffix of the long allele.
2. The first base pairs of all alleles are the same and the last base pairs of all alleles are the same.
3. Among all representations satisfying the previous two requirements, the given one is the shortest.

**Figure 1.**
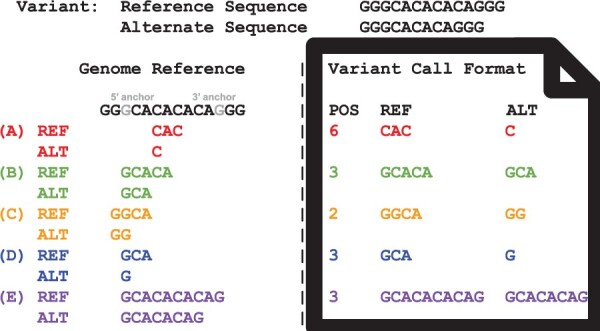
Example of indel VCF records representing the same variant with record (A) not left-aligned but parsimonious, record (B) left-aligned but not parsimonious, record (C) not left-aligned and not parsimonious, record (D) normalized, and record (E) maximally extended. Extended from previous work defining normalized VCF records (Tan *et al.* 2015).

Multiple VCF records can represent the same variant, while only one record can be normalized and only one record can be maximally extended (Fig. 1). Notice that normalized VCF records for SNVs are not maximally extended as the normalized representation does not satisfy the second requirement. Given an algorithm for computing a normalized VCF record, a VCF record can be maximally extended by the procedure described in Algorithm 1.

Algorithm 1 Maximally extend a VCF record

 **Input** A VCF record and the reference genome sequence

 **Output** A maximally extended VCF record

1: normalize the VCF record

2: **if** alleles do not all start with the same nucleotide **then**

3:  extend all alleles by 1 nucleotide to the left

4: **end if**

5: **if** alleles do not all end with the same nucleotide **then**

6:  extend all alleles by 1 nucleotide to the right

7: **end if**

8: **for** each pair of alleles **do**

9:  **while** the short allele is equal to the prefix or suffix of the longer allele **do**

10:   extend all alleles by 1 nucleotide to the right

11:  **end while**

12: **end for**

13: return the VCF record

For a given maximally extended record, we define the shared left-most base in the genome assembly as the 5’ anchor and the shared right-most base in the genome assembly as the 3’ anchor (Fig. 1).

To map base pairs from one genome assembly to another, all liftover tools require a chain file. A chain is a pairwise alignment between two DNA sequences that allows gaps in both sequences. A chain file used for liftover (extension .over.chain.gz) is a collection of non-overlapping chains encoded in the chain format (http://genome.ucsc.edu/goldenPath/help/chain.html) which has every base pair in the source assembly either not mapping to the destination assembly or mapping to a unique position in the target assembly. Chain files are generated from pairwise sequence alignments further filtered to provide unique coverage of the source assembly (Kent *et al.* 2003) (http://genomewiki.ucsc.edu/index.php/Chains_Nets).

### 2.2 Mapping strategy

For a given base pair in the old assembly, the tool maps the location in the new assembly using the regidx API for fast region lookup (Bonfield *et al.* 2021) to find overlaps with chain blocks defined in the input chain file.

**Figure 2.**
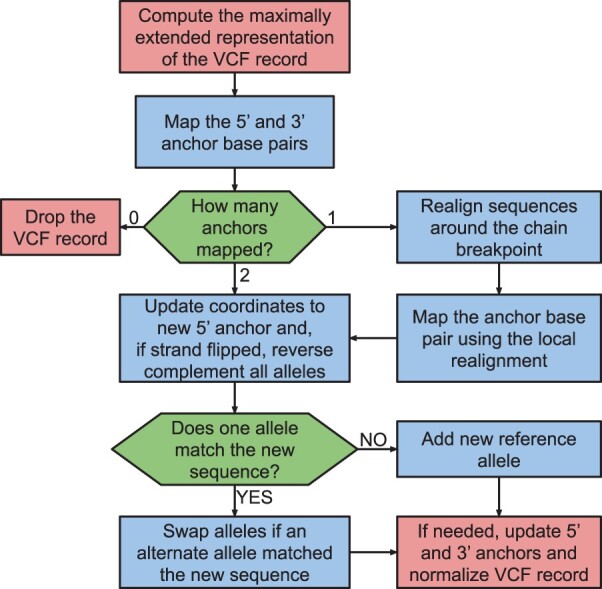
Strategy for the liftover process of a VCF record through the mapping of the 5’ and 3’ anchors of its maximally extended representation.

**Figure 3.**
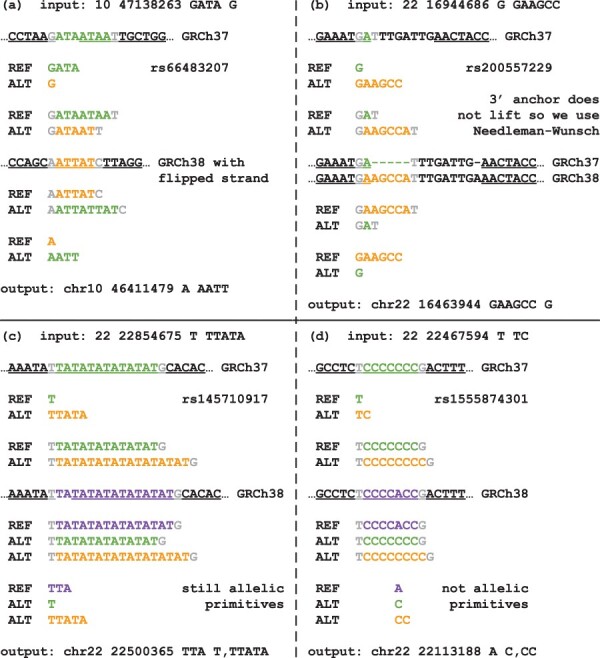
Examples of different liftover scenarios for indels: (a) a strand change combined with a reference and alternate allele swap; (b) a chain gap causing the 3’ anchor to fail to map to the new assembly requiring local realignment to find the most likely location of the anchor in the new assembly; (c) an STR liftover where neither allele matches the new assembly sequence due to a different length of the STR in the new assembly; and (d) an STR liftover where neither allele matches the new assembly sequence due to a SNV variation within the STR region itself leading to a multi-allelic record that is not an allelic primitive variant. Underlined base pairs are base pairs covered by the hg19ToHg38.over.chain.gz chain file. Gray base pairs are 5’ and 3’ anchors for the maximally extended representations of the records. Transanno/liftvcf correctly processes (a) and (c), fails to swap alleles in (b), and yields record chr22 22113183 T TC for (d), while Genozip/DVCF correctly processes (b), but drops (a), (c), and (d). Notice that variant rs1555874301 (d) is represented by VCF record chr22 22113183T TC in the high-coverage 1000 Genomes project, but it is not possible, without sequence context, to correctly convert this variant from GRCh37 to GRCh38.

For indel records, we always map the genomic location of the 5’ and 3’ anchors base pairs first and then we identify which of the reference or alternate alleles in the maximally extended representation matches the reference allele in the new assembly (Fig. 2), without requiring the base pairs of the anchors in the old assembly to also match the base pairs of the anchors in the new assembly and by reverse complementing the alleles if necessary (Fig. 3a). When an indel variant falls within the edge of a chain gap and we can only map the position of one of the two anchors, we map the position of the other anchor by locally realigning the sequence using an implementation of the Needleman-Wunsch algorithm with affine gap costs (Gotoh 1982) to identify the most likely location of the anchor that failed to map to the new assembly (Fig. 3b). This combined strategy correctly handles STR loci where the new assembly does not match any of the original reference and alternate alleles (Fig. 3c). In some rare cases, it adds a novel reference allele that when combined with one of the alternate alleles is neither a SNV nor an indel (Fig. 3d).

**Figure 4.**
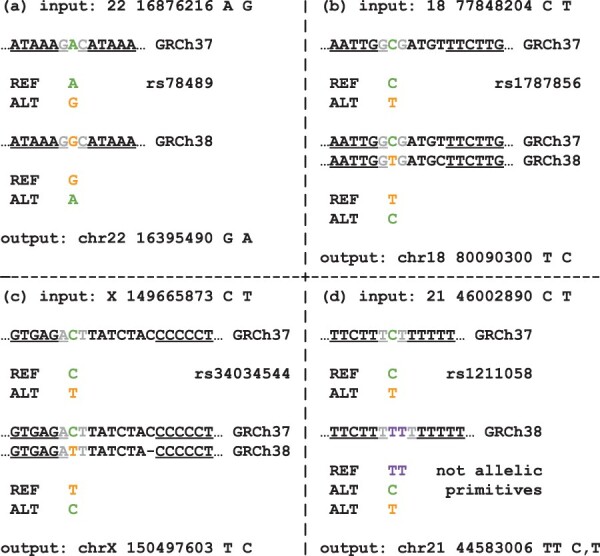
Examples of different liftover scenarios for SNVs from the low-coverage 1000 Genomes project falling within one of the chain gaps where the gap is caused by: (a) a SNV, (b) two SNVs, (c) a SNV and an indel, or (d) a complex variant. Underlined base pairs are base pairs covered by the hg19ToHg38.over.chain.gz chain file. Gray base pairs are 5’ and 3’ anchors for the maximally extended representations of the records. Transanno/liftvcf correctly processes (a), drops (b) and (c), and yields an incorrect record chr21 44583006T TT, TC for (d), while Genozip/DVCF, Picard/LiftoverVcf, and CrossMap/VCF drop all the SNVs. Notice that variant rs1211058 (d) is represented by VCF record chr21 44583007T C in the high-coverage 1000 Genomes project, but it is not possible, without sequence context, to correctly convert this variant from GRCh37 to GRCh38.

For SNV records, we simply convert the genomic location of the polymorphic base pair. If this base pair is not covered by the chain file, then we use the same strategy devised for indels based on mapping the 5’ and 3’ anchors of the maximally extended representation. This allows the tool to recover SNVs falling in gaps of the chain due to the new assembly sequence representing an alternative allele (Fig. 4a) and gaps caused by more than one allelic difference between the assemblies (Fig. 4b and c) that would otherwise be rejected by the other tools. This strategy occasionally leads to adding a reference allele that has a length longer than one base pair, leading to a new variant that is neither a SNV nor an indel (Fig. 4d).

SNVs and indels are defined as allelic primitives. Variants that are not allelic primitive variants are defined as complex, which includes variants such as multi-nucleotide variants (MNVs). Complex variants are allowed by the VCF specification, but they can always be split as a combination of allelic primitives, sometimes in multiple ways. Variants called from next-generation sequencing reads using the GATK HaplotypeCaller (Poplin *et al.* 2017) are always exclusively allelic primitives. When by converting the genomic coordinates of an allelic primitive record we obtain something that is not an allelic primitive record (Figs 3d and 4d), we cannot expect that such a variant would be able to match variants natively called by aligning the sequencing reads against the novel assembly. Therefore, for most practical purposes we can consider these records as if they were dropped during the conversion.

### 2.3 Tools comparisons

To compare the performance of BCFtools/liftover with Transanno/liftvcf, Genozip/DVCF, GenomeWarp, Picard/LiftoverVcf, and CrossMap/VCF, we ran each tool on 1000 Genome project variant callsets (Table 1) enriched for common variants as the more polymorphic a variant is the more likely it will be represented by different alleles across genome assemblies and therefore present additional challenges for conversion that would be unlikely to be encountered when converting rare variants. For a liftover from GRCh37 to GRCh38, we used variants identified in the low-coverage 1000 Genomes project (1000 Genomes Project Consortium 2015) together with the UCSC chain file (hg19ToHg38.over.chain.gz) generated using the same species protocol (http://genomewiki.ucsc.edu/index.php/DoSameSpeciesLiftOver.pl) from BLAT alignments (Kent 2002). For a liftover from GRCh38 to either the T2T-CHM13v2.0 or the Clint_PTRv2 assembly, the latest available chimpanzee assembly, we used variants identified in the high-coverage 1000 Genomes project (Byrska-Bishop *et al.* 2022) together with the UCSC chain files (either hg38ToHs1.over.chain.gz or hg38ToPanTro6.over.chain.gz) generated using the different species protocol (http://genomewiki.ucsc.edu/index.php/DoBlastzChainNet.pl) from LASTZ alignments (Harris 2007). For the liftover from GRCh37 to GRCh38, we did not use the Ensembl chain file (GRCh37_to_GRCh38.chain.gz) generated from Ensembl assembly mappings (http://github.com/Ensembl/ensembl/) as this resulted in a much higher rate of variants dropped compared with using the UCSC chain file. For the liftover from GRCh38 to the T2T-CHM13v2.0 assembly, we did not use the nf-LO (Talenti and Prendergast 2021) chain file (hg38-chm13v2.over.chain.gz) generated from minimap2 (Li 2018) alignments (http://github.com/marbl/CHM13\#liftover-resources) as this was affected by a bug in the chaintools software (Rhie *et al.* 2023) that collapsed double-sided gaps into single-sided gaps leading to erroneous mappings.

**Table 1.** Description of the two 1000 Genome project non-singleton variants callsets used for running genomic coordinates conversion comparisons and description of the three chain files used for the liftover conversion.

1000 Genomes	low coverage	high coverage
Project callset		
Release year	2013	2020
Number of samples	2504	3202
Variant calling	multiple callers	HaplotypeCaller
Aligned against	GRCh37	GRCh38
Sequencing coverage	7.4×	34×
Number of non-singleton variants		
SNVs	45 595 458	63 993 411
Bi-allelic indels	3 398 818	9 459 059
Multi-allelic indels (split)	243 179	4 123 095
Multi-allelic indels (merged)	108 842	1 375 718
Non-allelic primitives	1408	0
Chain properties		
Source assembly	GRCh37	GRCh38
Destination assembly	GRCh38	T2T-CHM13v2.0
		or Clint_PTRv2
Generation script	DoSameSpecies-	DoBlastz-
	LiftOver.pl	ChainNet.pl
Assembly aligner	BLAT	LASTZ
Chain file	hg19ToHg38	hg38ToHs1
(.over.chain.gz)		or hg38ToPanTro6

As the high-coverage callset used exclusively the HaplotypeCaller to call variants and used longer sequencing reads, an order of magnitude more multi-allelic indels are included and non-allelic primitive variants are not present in the callset.

We evaluated the performance of each tool for bi-allelic SNVs and bi-allelic indels separately. We further evaluated BCFtools/liftover on multi-allelic indels by joining bi-allelic indels at the same positions using BCFtools/norm run with option --multiallelics +. We made every effort to compare the tools in a consistent way. We run Transanno/liftvcf with option --no-left-align-chain to avoid the provided chain files losing their 1-to-1 mapping properties. Since Genozip/DVCF automatically compresses the input VCF and we were only interested in its liftover capabilities, we ran the tool with options --fast and --vblock 1 to minimize the time spent for the compression. We ran GenomeWarp with option --keep_homozygous_reference_calls, and we ran Picard/LiftoverVcf with option --RECOVER_SWAPPED_REF_ALT true. We further ran GenomeWarp and Picard/LiftoverVcf with options, respectively, --min_match 0.0 and --LIFTOVER_MIN_MATCH 0.0 to maximize the number of converted indel records. As BCFtools/liftover, Transanno/liftvcf, and CrossMap/VCF do not sort the output, while Genozip/DVCF and Picard/LiftoverVcf do, we ran BCFtools/sort with option --max-mem 128M on the latter tools’ output to properly compare the speed of each tool. While BCFtools/liftover, Transanno/liftvcf, and Picard/LiftoverVcf left-align the output, but Genozip/DVCF and CrossMap/VCF do not, we further ran BCFtools/norm on the latter tools’ output. To measure which output records were not allelic primitive variants, we used BCFtools/view with option --types mnps, other. We notice that these analyses do not attempt to recover non-polymorphic variants represented by different alleles across the two assemblies and which would have been missing from the input callset if all samples in the cohort had homozygous reference genotypes for that variant, something that GenomeWarp was designed to handle instead (McLean *et al.* 2019).

## 3 Results

### 3.1 Conversion to canonical human genome assembly

**Figure 5.**
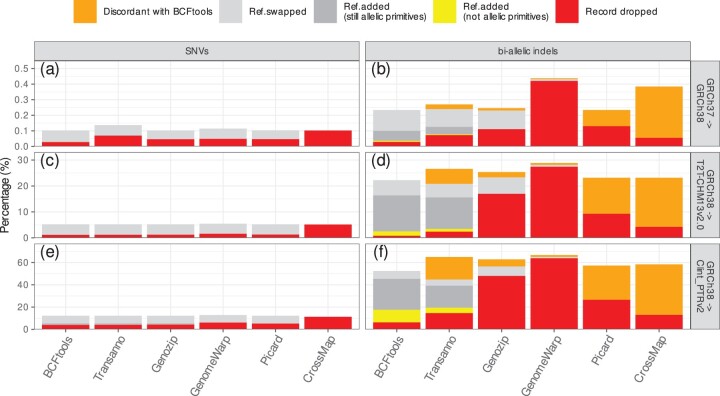
VCF liftover tools comparison between BCFtools/liftover and five available VCF liftover tools across six different scenarios: (a) SNVs from GRCh37 to GRCh38; (b) bi-allelic indels from GRCh37 to GRCh38; (c) SNVs from GRCh38 to T2T-CHM13v2.0; (d) bi-allelic indels from GRCh38 to T2T-CHM13v2.0; (e) SNVs from GRCh38 to Clint_PTRv2; and (f) bi-allelic indels from GRCh38 to Clint_PTRv2. Bar graph reports fractions of VCF records from the 1000 Genomes project dropped, with a reference allele added either leading or not leading to an allelic primitive variant, or with the reference allele swapped with one of the alternate alleles, or discordant with the output of BCFtools/liftover. BCFtools/liftover has the lowest rate of SNVs and indels dropped.

When converting VCF records from GRCh37 to GRCh38, we expect few variants to drop or change reference allele as the two genome assemblies are mostly identical with differences restricted to complex regions that were revised and updated. When processing variants from the low-coverage 1000 Genomes project, we notice that all tools except CrossMap/VCF handle SNVs in similar ways (Fig. 5a). Out of a total of 45 595 458 bi-allelic SNVs, Transanno/liftvcf drops 31 436 SNVs, mostly complaining that the majority mapped to multiple regions, Genozip/DVCF, GenomeWarp, and Picard/LiftoverVcf drop, respectively, 21 068, 22 251, and 21 503 SNVs, and CrossMap/VCF, as it is unable to swap the reference and alternate alleles, drops a total of 46 607 SNVs. Conversely, BCFtools/liftover only drops 12 582 SNVs as almost half of the SNVs dropped by Genozip/DVCF, GenomeWarp, and Picard/LiftoverVcf fall in either one base pair chain gaps (Fig. 4a) or other gaps caused by a pair of variants both represented by different alleles between the two assemblies (Fig. 4b and c) in the UCSC chain file which can be properly handled by BCFtools/liftover.

When converting bi-allelic indels from GRCh37 to GRCh38, we again observe that BCFtools/liftover has the lowest dropping rate. Out of a total of 3 398 818 bi-allelic indels, BCFtools/liftover drops 999 indels, compared to 2462 for Transanno/liftvcf, 3785 for Genozip/DVCF, 4429 for Picard/LiftoverVcf, and 1885 for CrossMap/VCF (Fig. 5b). GenomeWarp drops 14 307 bi-allelic indels as it is deliberately conservative in difficult cases. BCFtools/liftover has also the highest rate of swapped indel alleles at 4562, compared to 3898 for Transanno/liftvcf, 4074 for Genozip/DVCF, and 379 for GenomeWarp, while Picard/LiftoverVcf and CrossMap/VCF cannot perform swaps when it comes to indels. BCFtools/liftover further adds a reference allele to 2385 bi-allelic indels. We find that for 2210 of these, the resulting multi-allelic record is a multi-allelic STR record (Fig. 3c), while for 235 of these, the output is not an allelic primitive variant (Fig. 3d). Even if we include these 235 cases as failures the overall drop rate of BCFtools/liftover is still lower than the one of all the other tools. Similarly, Transanno/liftvcf, the only other tool capable of increasing the number of alleles, does so for 1796 indels records with the result that in 152 cases the resulting multi-allelic record is not an allelic primitive record.

**Figure 6.**
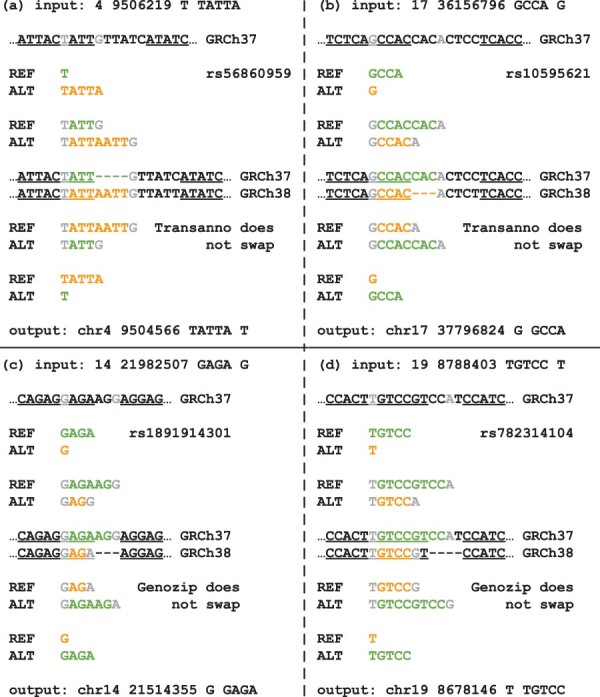
Examples of different liftover scenarios with discordance between BCFtools/liftover and either Transanno/liftvcf (a, b) or Genozip/DVCF (c, d) where the latter tools are unable to recognize the need for a swap of the reference with the alternate allele in the indel record. In each scenario, the 3’ anchor fails to map to the new assembly due to a small gap in the chain outlining the alignment between the two assemblies. BCFtools/liftover uses the Needleman-Wunsh algorithm to realign the sequence overlapping the gap and assess which base pair in the new assembly should act as a 3’ anchor. Underlined base pairs are base pairs covered by the hg19ToHg38.over.chain.gz chain file. Gray base pairs are 5’ and 3’ anchors for the maximally extended representations of the records.

As Picard/LiftoverVcf and CrossMap/VCF have no implemented capability to swap reference and alternate alleles or add a reference allele when processing indel records, when comparing the output for bi-allelic indel records with BCFtools/liftover output, we find 3523 discordant records for Picard/LiftoverVcf and 11 184 discordant records for CrossMap/VCF. Conversely for Transanno/liftvcf and Genozip/DVCF, we only find, respectively, 1007 and 503 discordant records (Fig. 5b), and with Genomewarp we only find 196 discordant records as the tool is deliberately conservative in complex cases. For Transanno/liftvcf, in 743 cases only one tool added a reference allele to the VCF record, in 190 cases only one tool swapped reference and alternate alleles (Fig. 6a and b), in 61 cases each tool added a different reference allele, and in 13 cases the two tools mapped the records to different base pairs. As Genozip/DVCF and GenomeWarp cannot add reference alleles to a VCF record, for Genozip/DVCF 424 discrepancies are cases where BCFtools/liftover added a reference allele and 79 discrepancies are cases where only one tool swapped reference and alternate alleles (Fig. 6c and d), while for GenomeWarp 157 discrepancies are cases where BCFtools/liftover added a reference allele and 39 discrepancies are cases where only one tool swapped reference and alternate alleles.

### 3.2 Conversion to telomere-to-telomere human genome assembly

When converting VCF records from GRCh38 to T2T-CHM13v2.0, we expect a large fraction of commonly polymorphic variants to change reference allele as the two genome assemblies represent completely different human genome haplotypes. When processing variants from the high-coverage 1000 Genomes project, we again notice that all tools except CrossMap/VCF handle SNVs in similar ways (Fig. 5c) with BCFtools/liftover dropping 741 574 SNVs out of a total of 63 993 411 bi-allelic SNVs, while Transanno/liftvcf, Genozip/DVCF, GenomeWarp, and Picard/LiftoverVcf drop, respectively, 757 454, 769 903, 1 033 949, and 829 097 SNVs. As CrossMap/VCF is unable to perform allele swaps, it drops 3 285 604 SNVs.

When converting bi-allelic indels from GRCh38 to T2T-CHM13v2.0 (Fig. 5d), out of 9 459 059 bi-allelic indels, BCFtools/liftover drops 78 119 indels and adds a reference allele so that the new record is not an allelic primitive variant for 156 233 records. By comparison, Transanno/liftvcf drops 228 660 indels and produces 98 248 records that are not allelic primitive variants, while Genozip/DVCF and GenomeWarp drop, respectively, 1 606 397 and 2 594 586 records. Picard/LiftoverVcf and CrossMap/VCF, which are unable to swap or add alleles when converting indel records, drop, respectively, 879 552 and 403 553 indels.

Out of 9 459 059 bi-allelic indel records, 4 123 095 can be merged into 1 375 718 multi-allelic indel records (Table 1). For this subset of records, the number of cases when a reference allele is added decreases from 1 167 101 to 172 091 reflecting that for many indels it is more appropriate to join indels at the same loci before performing the conversion to avoid multiple instances of the same reference allele being added across different VCF records.

### 3.3 Conversion to genome assembly from closely related species

When converting VCF records from GRCh38 to Clint_PTRv2, the latest available chimpanzee assembly, we expect an even larger fraction of polymorphic variants to change reference allele as the reference genome assembly of a closely related species will often be represented by the ancestral allele at the location corresponding to the polymorphic locus. Out of 63 993 411 bi-allelic SNVs BCFtools/liftover, Transanno/liftvcf, Genozip/DVCF, GenomeWarp, Picard/LiftoverVcf, and CrossMap/VCF drop, respectively, 2 525 783, 2 564 668, 2 683 616, 3 931 981, 3 365 790, and 7 163 292 variants (Fig. 5e) and out of 9 459 059 bi-allelic indels, they drop, respectively, 596 821, 1 387 488, 4 542 500, 6 048 367, 2 516 790, and 1 233 403 variants (Fig. 5f). BCFtools/liftover and Transanno/liftvcf produce, respectively, 1 059 460 and 445 898 indel records that are not allelic primitive variants. Picard/LiftoverVcf, and CrossMap/VCF yield discordant results with BCFtools/liftover for, respectively, 2 904 565 and 4 291 070 indel records. Overall each tool drops at least close to 4% of all SNVs and either drops or produces non-allelic primitives for more than 15% of all bi-allelic indels. However, between dropped and discordant records, GenomeWarp, Picard/LiftoverVcf, and CrossMap/VCF fail to convert more than 50% indel records, compared to BCFtools/liftover and Transanno/liftvcf that do so for less than 20% indel records, highlighting how the former tools are not designed to handle complicated indels scenarios.

### 3.4 Update of variants annotations

While conversion of chromosome, position, allele fields, and genotypes from a VCF record is the most important task of the liftover process, there are additional features relevant in a conversion. Compared to the other tools, BCFtools/liftover supports the largest number of features while between Transanno/liftvcf, Genozip/DVCF, GenomeWarp, Picard/LiftoverVcf, and CrossMap/VCF, we find that Transanno/liftvcf has the most features and CrossMap/VCF has the least (Table 2). As BCFtools/liftover is the only tool built on top of BCFtools (Danecek *et al.* 2021) and HTSlib (Bonfield *et al.* 2021), which provide input/output capabilities, it is also the only tool that can handle binary VCF records.

**Table 2.** Comparison of features and limitations across BCFtools/liftover and five available VCF liftover tools.

Tool	BCFtools	Transanno	Genozip	GenomeWarp	Picard	CrossMap
	liftover	liftvcf	DVCF		LiftoverVcf	VCF
Version tested	2023-12-06	0.4.4	15.0.27	1.1.0	3.1.1	0.6.6
GitHub username	freeseek	informationsea	divonlan	verilylifesciences	broadinstitute	liguowang
GitHub repository	score	transanno	genozip	genomewarp	picard	CrossMap
License	MIT	GPLv3	proprietary	Apache	MIT	GPLv3
Main features						
Can reverse-complements alleles	Yes	Yes	Yes	Yes	Yes	Yes
Handles multi-allelic records	Yes	Yes	No	No	No	No
Can swap SNV alleles	Yes	Yes	Bi-allelic only	Bi-allelic only	Bi-allelic only	No
Can swap indel alleles	Yes	Yes	Bi-allelic only	Bi-allelic only	No	No
Can add new reference allele	Yes	Yes	No	SNVs only	No	No
Can recover SNVs at chain gaps	Yes	Yes	No	No	No	No
File input/output options						
Sort records after liftover	No	No	Yes	No	Yes	No
Left-aligns indels after liftover	Yes	Yes	Left-anchors	Yes	Yes	No
Can record the original position	Yes	Yes	Yes	No	Yes	No
Flexible with contig names	Yes	No	Yes	No	No	Yes
Loads full reference in memory	No	No	Yes	Yes	Yes	No
Can input VCF as a file stream	Yes	Yes	Yes	No	No	Yes
Can output VCF as a file stream	Yes	Yes	No	No	No	No
Can input/output binary VCFs	Yes	No	No	No	No	No
Variants annotations updates						
Updates INFO/END field	Yes	No	Yes	No	No	No
Updates Number=G/R fields	Yes	R records	Common ones	No	PL and AD	No
Updates AC-like fields	Yes	Yes	Yes	No	No	No
Updates AF-like fields	Yes	Yes	Yes	No	Yes	No
Updates GWAS-VCF fields	Yes	No	Yes	No	No	No

BCFtools/liftover has the largest number of features.

When the order of the alleles in a VCF record changes as a result of the liftover process, either because reference and alternate alleles are swapped or because a new reference allele is introduced, fields with one value per allele (Number=R) and fields with one value per genotype (Number=G) are automatically re-ordered by BCFtools/liftover. Other fields with one record per alternate allele (Number=A) are also updated according to specific rules. For example, VCF fields for the allele frequency (AF) are reordered by keeping the assumption that the sum of the values across all alleles, including the reference allele, is 1. Similarly, VCF fields for the allelic count in genotypes (AC) are reordered assuming that the sum of the values across all alleles is the total number of alleles in called genotypes (AN). When the reference and alternate alleles are swapped, the signs of the corresponding VCF fields for the effect size (ES) and for the Z-score (EZ) from the GWAS-VCF specification (Lyon *et al.* 2021) are reversed. While Transanno/liftvcf, Genozip/DVCF, and Picard/LiftoverVcf were capable of some of these updates, BCFtools/liftover supported the most updates (Table 2).

### 3.5 Speed and memory consumption

**Figure 7.**
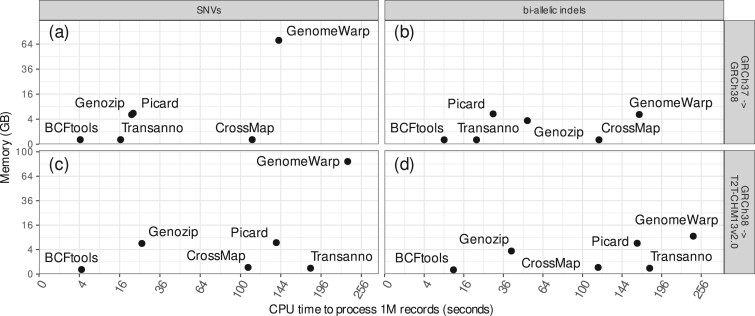
Speed and memory comparison between BCFtools/liftover and five available VCF liftover tools across four different scenarios: (a) SNVs from GRCh37 to GRCh38, (b) bi-allelic indels from GRCh37 to GRCh38, (c) SNVs from GRCh38 to T2T-CHM13v2.0, and (d) bi-allelic indels from GRCh38 to T2T-CHM13v2.0. BCFtools/liftover is the fastest tool with negligible memory requirements.

BCFtools/liftover is the fastest tool, taking an average of approximately 4 s to process one million SNVs (Fig. 7a and c) and 10 s to process one million bi-allelic indels (Fig. 7b and d) on a single CPU core. All other tools are at least four times slower to process SNVs and two times slower to process indels with GenomeWarp and CrossMap/VCF more than ten times slower. We also notice that while BCFtools/liftover, Transanno/liftvcf, and CrossMap/VCF have negligible memory requirements, Genozip/DVCF, GenomeWarp, and Picard/LiftoverVcf all require the whole human genome assembly to be loaded into memory, regardless of the number of records to be processed. GenomeWarp memory requirements increase with the number of records, making it unable to process large VCFs unless the user manually splits them into smaller files first.

## 4 Discussion

BCFtools/liftover is an accurate and comprehensive tool to convert the genomic coordinates of VCF records from large cohorts which outperforms any other same-purpose tool available at the time of the writing of this manuscript with significant improvements for the proper handling of indels and multi-allelic variants when compared to other tools commonly used for the same task such as Picard/LiftoverVcf and CrossMap/VCF. As BCFtools/liftover can effectively work around small alignment gaps between two assemblies, large regions of one assembly that are not included or represented in the other assembly, rather than allelic variation between the two assemblies largely expected with the new telomere-to-telomere assemblies (Aganezov *et al.* 2022, Nurk *et al.* 2022, Rhie *et al.* 2023), are left as the main limitations of the liftover process.

Regardless of the increased accuracy of BCFtools/liftover compared to the other tools, we warn that the liftover process is in general a lossy procedure and should not be regarded as a substitute for realigning sequences against a different genome assembly for datasets with available raw data as realignment and re-calling of variants will always generate better results (Zheng-Bradley *et al.* 2017, Lowy-Gallego *et al.* 2019). Similarly, for DNA microarray datasets with available raw data, we recommend realigning the manifest files with BCFtools/gtc2vcf (http://github.com/freeseek/gtc2vcf). For all scenarios where accessing the raw data is not feasible, e.g. GWAS summary statistics for legacy genome assemblies, BCFtools/liftover will handle the conversion of the coordinate system while reducing artifacts that could lead to biases in downstream analyses.

Finally, by adding to a growing family of easy-to-use tools for annotation (Danecek and McCarthy 2017), query, and normalization of VCF records, BCFtools/liftover greatly reduces the efforts needed to harmonize existing resources and accelerate the adoption of the GWAS-VCF standard (Lyon *et al.* 2021) to encode GWAS summary statistics by encouraging other developers to support this format and thus simplifying the task of running meta-analyses and computing polygenic scores.

## Data Availability

Source code is available at http://github.com/freeseek/score.
